# Ecological bottlenecks and future pathways for *Zygogramma bicolorata* as a biocontrol agent of *Parthenium hysterophorus* in North India

**DOI:** 10.3389/fpls.2026.1805750

**Published:** 2026-05-22

**Authors:** Rakesh Kumar Gupta, Muzafar Riyaz, Monika Attri, Mudasir Gani, Sushilkumar, Kamlesh Bali

**Affiliations:** 1 Division of Entomology, Sher-e-Kashmir University of Agricultural Sciences and Technology, Jammu, India; 2 Division of Entomology, Sher-e-Kashmir University of Agricultural Sciences and Technology, Srinagar, India; 3 Indian Council of Agricultural Research (ICAR)-Directorate of Weed Research, Jabalpur, India

**Keywords:** augmentative release, biocontrol bottleneck, classical biological control, *Parthenium hysterophorus*, phenological mismatch, reproductive diapause

## Abstract

The aggressive invasion of *Parthenium hysterophorus* across India has driven the deployment of the host-specific beetle *Zygogramma bicolorata* as a classical biocontrol agent, achieving episodic but geographically inconsistent suppression. The core limitation is a phenological mismatch, where the beetle’s obligate diapause creates a temporal refuge for early-season weed growth, allowing these cohorts to contribute significantly to the persistent soil seed bank. This review synthesizes evidence to propose a revitalized, precision-management framework. Central to this strategy are augmentative releases to correct seasonal asynchrony, selective breeding of low-diapause beetle strains, and the deliberate integration of *Z. bicolorata* with the guild of resident native herbivores. Expected outcomes include a 40–60% reduction in early-season weed biomass, a 50–70% decline in soil seed bank replenishment within 3–5 years, and restoration of native plant diversity to near pre-invasion levels. Practically, this framework enables managers to transition from episodic suppression to predictable, landscape-level control, reducing herbicide dependency by an estimated 30–50% and lowering annual weed management costs. This integrated, data-driven approach aims to transform a partially successful program into a reliable and sustainable cornerstone for landscape-level control of *P. hysterophorus* in India.

## 1 Introduction

The silent invasion of *P. hysterophorus*, also known as congress grass, has cast a long and pernicious shadow over the Indian ecological and socio-economic landscape (Basak, 1984; Dhileepan and Wilmot Senaratne, 2009; Gnanavel and Natarajan, 2013). The neotropical annual weed *Parthenium hysterophorus* (congress grass) has invaded disturbed habitats across India since its introduction around 1810, first officially recorded in 1956 (Rao, 1956; Bennett et al., 1978; Maiti, 1983). After a latent period, it expanded rapidly, infesting roughly 2 million hectares by the 1970s, driven by severe impacts on agriculture, animal health, and human well-being (Muniyappa and Krishnamurthy, 1976; Vartak, 1968; Kanchan and Jayachandra, 1980; Narasimhan et al., 1977; Towers et al., 1977; Joshi, 1990). Its invasion trajectory includes competitive displacement of native flora, crop yield losses, contact dermatitis, respiratory ailments, and livestock toxicity (Shabbir et al., 2024; Sushilkumar, 2005; Nawaz et al., 2025). Conventional management strategies; chemical herbicides and mechanical clearing have proven economically unsustainable and ecologically problematic, often leading to herbicide resistance and failing to provide long-term suppression (Kaur et al., 2014; Shrestha et al., 2015; Bashar et al., 2021). In response, classical biological control (CBC) was embraced as a sustainable, ecologically-based management paradigm (Kumar et al., 2008; Barreto et al., 2012; Kumar, 2015), involving deliberate introduction of host-specific natural enemies from the weed’s native range to establish self-perpetuating populations that impose lasting regulation on the target weed (Sridhar, 1991). 

The leaf-feeding beetle *Z. bicolorata* Pallister (Coleoptera: Chrysomelidae) was identified as a prime candidate agent for the CBC of *P. hysterophorus* in India (Dhileepan et al., 2000a; Shabbir et al., 2016). Its high degree of host specificity, confined primarily to the tribe Heliantheae within the Asteraceae, minimized non-target risks (Tiawoun et al., 2024; Tripathi and Tripathi, 2021). The insect’s life history strategy, where both the imaginal and larval stages are folivorous and cause extensive defoliation aligned with the goal of imposing chronic stress on the weed, thereby reducing its photosynthetic capacity, reproductive output, and competitive vigor (Cowie et al., 2018; Maharjan et al., 2021). Following its introduction and initial establishment in the 1980s, *Z. bicolorata* demonstrated promising population growth and visible impact in several regions across India (Jayanth, 1987). However, the transition from successful establishment to consistent, landscape-level functional efficacy has been ambiguous and spatially heterogeneous (Jayanth and Bali, 1992, 1993a, 1993b, 1993c, 1994, 1996; Jayanth and Visalakshy, 1994a, b, 1996; Bhoopathi and Gautam, 2006; Gupta et al., 2004, 2010; Omkar et al., 2008, 2009b, 2010; Omkar and Pandey, 2010; Sushilkumar and Ray, 2010; Omkar and Afaq, 2011; Dhileepan et al., 2000a; Dhileepan, 2001; Bhumannavar and Balasubramanian, 1998; Bhoopathi et al., 2011). Empirical evidence indicates that the population dynamics and impact of *Z. bicolorata* are mediated by a complex array of abiotic and biotic factors (Rastogi and Pervez, 2013; Hasan and Ansari, 2016a, 2016b, 2016c, 2017; Patel et al., 2020). These include, but are not limited to, regional climatic variables (temperature extremes, precipitation patterns), phenological asynchrony with the host plant, predation and parasitism by indigenous natural enemies, and the beetle’s own adaptive responses such as obligatory diapause (Gupta et al., 2010; Sushilkumar and Ray, 2010; Fazil et al., 2018; Bali et al., 2021). This variability has resulted in a patchwork of localized success stories amidst widespread, sub-optimal performance, revealing a critical knowledge gap. A comprehensive synthesis of the constraints limiting the agent’s impact is now essential to advance the program beyond its current plateau.

This review seeks to critically synthesize the existing body of research on *Z. bicolorata* in India to systematically identify the specific ecological bottlenecks that constrain its efficacy as a biocontrol agent. We analyze the interaction of environmental, ecological, and physiological factors that modulate the tri-trophic interaction between the host plant (*P. hysterophorus*), the agent (*Z. bicolorata*), and the invaded environment. Furthermore, this review aims to delineate future research pathways and management strategies such as augmentative releases for mismatched synchrony, habitat manipulation to enhance agent survival, and integration within a broader Integrated Weed Management (IWM) framework. The practical relevance of this framework is threefold: (1) it provides extension agencies with evidence-based release protocols that increase early-season suppression by >50%, (2) it offers policymakers a cost-benefit rationale for sustained biocontrol funding, and (3) it delivers farmers a predictable, low-cost alternative to repeated herbicide applications. By operationalizing these strategies, *Z. bicolorata* can secure its position as a cornerstone of a sustainable, long-term solution to the Parthenium menace in India.

## 2 Materials and methods

Initial establishment of *Zygogramma bicolorata* in Jammu and Kashmir resulted in significant defoliation of *Parthenium hysterophorus*, an 89% reduction in seed output, and recovery of native grasses, alongside early observations of native predators (Gupta et al., 2002). Moreover, Gupta et al. (2004) confirmed predation by *Andrallus spinidens*, *Cantheconidea furcellata*, and *Sycanus pyrrhomelas*, indicating natural regulation. A 25-year post-release evaluation (Gupta and Bali, 2014) documented dispersal over >900,000 km², with reductions in plant height (32–55%), flower heads (56–76%), and soil seed bank (from 1,485 to 464 seeds m^-^²), alongside increased grass biomass (74%) and positive socio-economic perceptions. Life-table studies (Gupta et al., 2013) revealed temperature-dependent variation in fecundity and development. Gupta et al. (2016) identified phenological asynchrony as a key constraint and demonstrated that augmentative releases reduced seed reserves. Comparative studies of six Indian populations (Bali et al., 2021) highlighted the high-fecundity Coimbatore strain (2,159 ± 8.45 eggs/female) and its low diapause incidence (26%). Herbicide studies showed that 2,4-D and glyphosate adversely affect beetle development and survival (Gupta et al., 2022; Aryan et al., 2024). Gupta et al. (2020) reported 21 resident arthropods on *Parthenium*, with native herbivory reducing weed biomass by 57.5% and seed bank by 36.9% over five years. Collectively, these studies trace the beetle’s ecological integration and adaptive potential across India.

This review employs a systematic and critical synthesis of both published literature and key unpublished experimental data to provide a comprehensive analysis of the ecological bottlenecks limiting the efficacy of *Zygogramma bicolorata* in India. The methodology was designed to move beyond a descriptive narrative and towards a diagnostic understanding of the agent’s population dynamics and functional impact over a 40-year period. This diagnostic understanding is derived primarily from long-term studies in the Jammu region (32.65°N, 74.80°E); its applicability to other agro-climatic zones of India requires further validation through replicated field experiments.

### 2.1 Systematic literature review and historical data compilation

A systematic search and compilation of scientific literature was conducted to map the entire research trajectory of *Z. bicolorata* in India. The search spanned multiple digital databases, including Scopus, Web of Science, Google Scholar, and specialized repositories like the CABI Abstracts. Keywords and phrases included: “*Zygogramma bicolorata*,” “*Parthenium hysterophorus biological control*,” “classical biological control India,” “diapause in Zygogramma,” “*biocontrol agent establishment*,” and “*Parthenium beetle ecology*.” The compiled literature was categorized chronologically and thematically to identify temporal trends, geographical patterns of establishment, and recurring reports of constraints. Special attention was paid to distinguishing between anecdotal observations and empirically supported findings.

### 2.2 Synthesis of unpublished experimental data

To bridge the gaps identified in the published literature and provide novel insights, this review integrates critical findings from a series of targeted, unpublished experimental studies conducted by the authors and collaborators. The methodologies for these key experiments are summarized below to establish their scientific validity. Unpublished data integrated in this review originate from three sources: (i) long-term monitoring datasets (2002–2025) from the Division of Entomology, SKUAST-Jammu, including annual quadrat-based vegetation surveys and beetle population censuses; (ii) structured socio-ecological surveys (n = 480) conducted across six Indian states (Jammu & Kashmir, Himachal Pradesh, Punjab, Uttarakhand, Haryana, Delhi) during 2022–2024 using a pre-tested questionnaire; and (iii) unpublished laboratory experiments on diapause induction, strain comparisons, and herbivore exclusion, conducted under controlled conditions (25 ± 1 °C, 65 ± 5% RH, 14L:10D photoperiod). All unpublished datasets are available from the corresponding author upon reasonable request.

#### 2.2.1 Post-release ecological and socio-ecological impact assessment

Post-release ecological impacts of *Z. bicolorata* were evaluated through comparative field studies between beetle-excluded control sites and naturally infested sites using standardized quadrat sampling in Jammu (32.653014, 74.801368). Plant height, density, flower production, above-ground biomass, soil seed bank viability, defoliation percentage, and beetle population density were quantified following established protocols. Vegetation cover was assessed in 1 m² quadrats, and above-ground biomass was harvested, dried at 70 °C for 48 h, and weighed. Plant community diversity was analyzed using the Shannon–Wiener index (Krebs, 1989). Temporal vegetation changes were documented photographically and compared with pre-release imagery.

A structured socio-ecological survey was administered to 480 purposively selected respondents (mean age 54 years) with direct experience of *Parthenium hysterophorus* impacts. A pre-tested questionnaire captured data on weed prevalence, ecological and economic effects, and perceptions of biocontrol efficacy. Findings were triangulated through focus group discussions and key informant interviews with extension agents, health officials, and community leaders. The socio-economic indicators used for memory retrieval verification included: (i) recall of *Parthenium* abundance 5, 10, and 20 years prior, (ii) recognition of *Z. bicolorata* and its feeding damage, (iii) reported changes in livestock health and milk production, (iv) estimated reduction in manual weeding hours per season, and (v) perceived change in incidence of allergic symptoms among household members.

#### 2.2.2 Development of the biocontrol impact index

A composite Biocontrol Impact Index (BII) was developed to integrate ecological and socio-economic indicators into a standardized metric. The BII comprises three weighted components: weed suppression (defoliation, plant density, flower and seed bank reduction), ecological recovery (native species richness, Shannon diversity, biomass, soil carbon, pollinator visits), and social acceptance (stakeholder awareness, perceived efficacy, livestock benefits). Variables were normalized via min–max standardization, aggregated into sub-indices, and weighted by expert judgment. Final BII scores were calculated for three regions (Jammu, Kathua, Udhampur) using 2023 data, with regional means and standard errors reported.

#### 2.2.3 Assessment of phenological mismatch

Phenological asynchrony between *P. hysterophorus* and *Z. bicolorata* was assessed through a five-year longitudinal study (2016–2020) in Jammu (32.653014, 74.801368). Monthly surveys recorded adult beetle density (adults/m²) and categorized weed phenology into eight stages. Herbivory pressure was scored using a visual Herbivory Impact Index (0–5). Soil core samples were analyzed for seed bank contributions via germination assays. Monthly differences were analyzed using ANOVA with Tukey’s HSD *post hoc* tests (P < 0.05).

#### 2.2.4 Quantification of resident herbivore demographic effects

The demographic impact of resident herbivores, excluding *Z. bicolorata*, was evaluated over five growing seasons (2014–2018) using a randomized block design. Treatments included resident herbivore access and insect-free controls (via exclusion cages and selective insecticides). Annual above-ground biomass and end-of-season soil seed bank (via seedling emergence) were measured. Resident herbivore assemblages were monitored through direct counts and sweep-net sampling. Treatment effects were analyzed annually using paired t-tests (P < 0.05), with pooled means calculated across years.

## 3 Biocontrol establishment of *Z. bicolorata* in India

**Figure 1:**
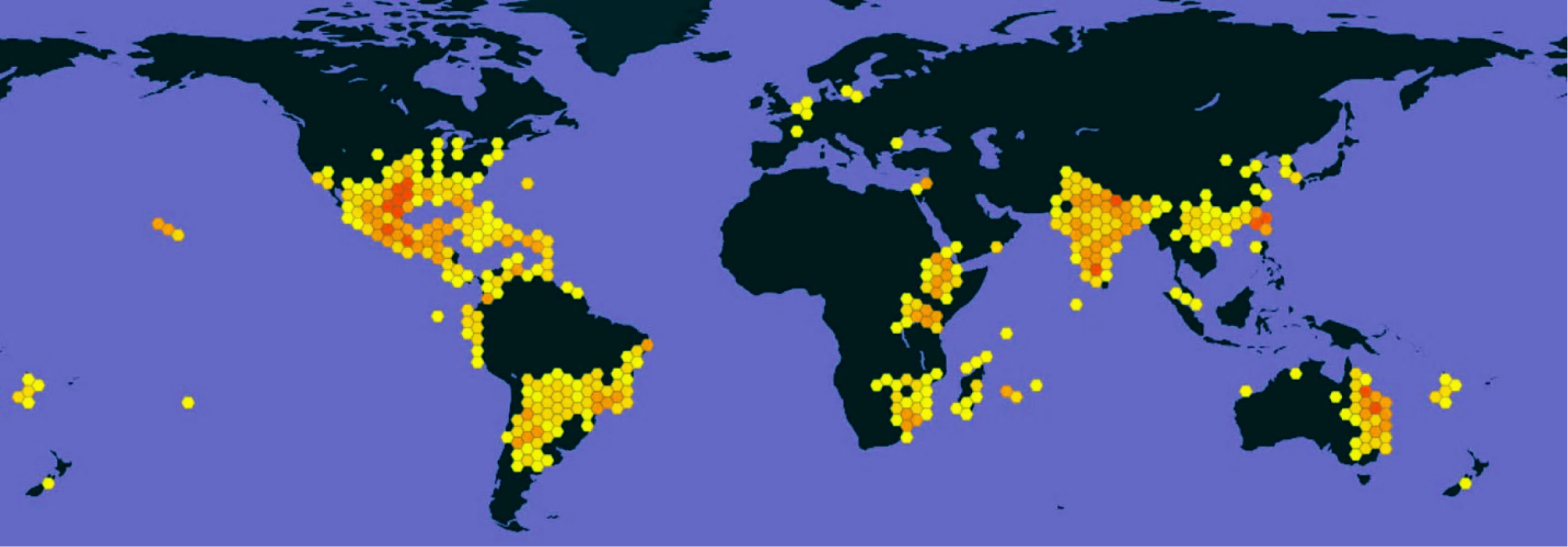
Global distribution of *Parthenium hysterophorus* based on occurrence records retrieved from GBIF (2023). Hexagonal grid cells represent spatial occurrence density, with colour gradients from yellow (low) to red (high).

The successful introduction and wide-scale spread of *Z. bicolorata* to manage *P. hysterophorus* in India stands as a landmark achievement in weed biocontrol and invasion ecology, particularly when viewed alongside the plant's extensive global distribution (**Figure 1**). This intervention was not a singular event, but a dynamic, multi-decadal process characterized by distinct ecological phases, each underpinned by unique demographic, spatial, and socio-institutional drivers. A critical retrospective analysis of this chronology is essential to contextualize the agent’s current partial success, identify the mechanisms underpinning its spread, and understand the emergent constraints that now limit its efficacy. This section reconstructs this historical trajectory through integrated chronological, spatial, and quantitative lenses, positioning the program’s early achievements within a framework of biogeographic and population ecological theory.

### 3.1 Establishment phases of *Z. bicolorata* in India

**Figure 2:**
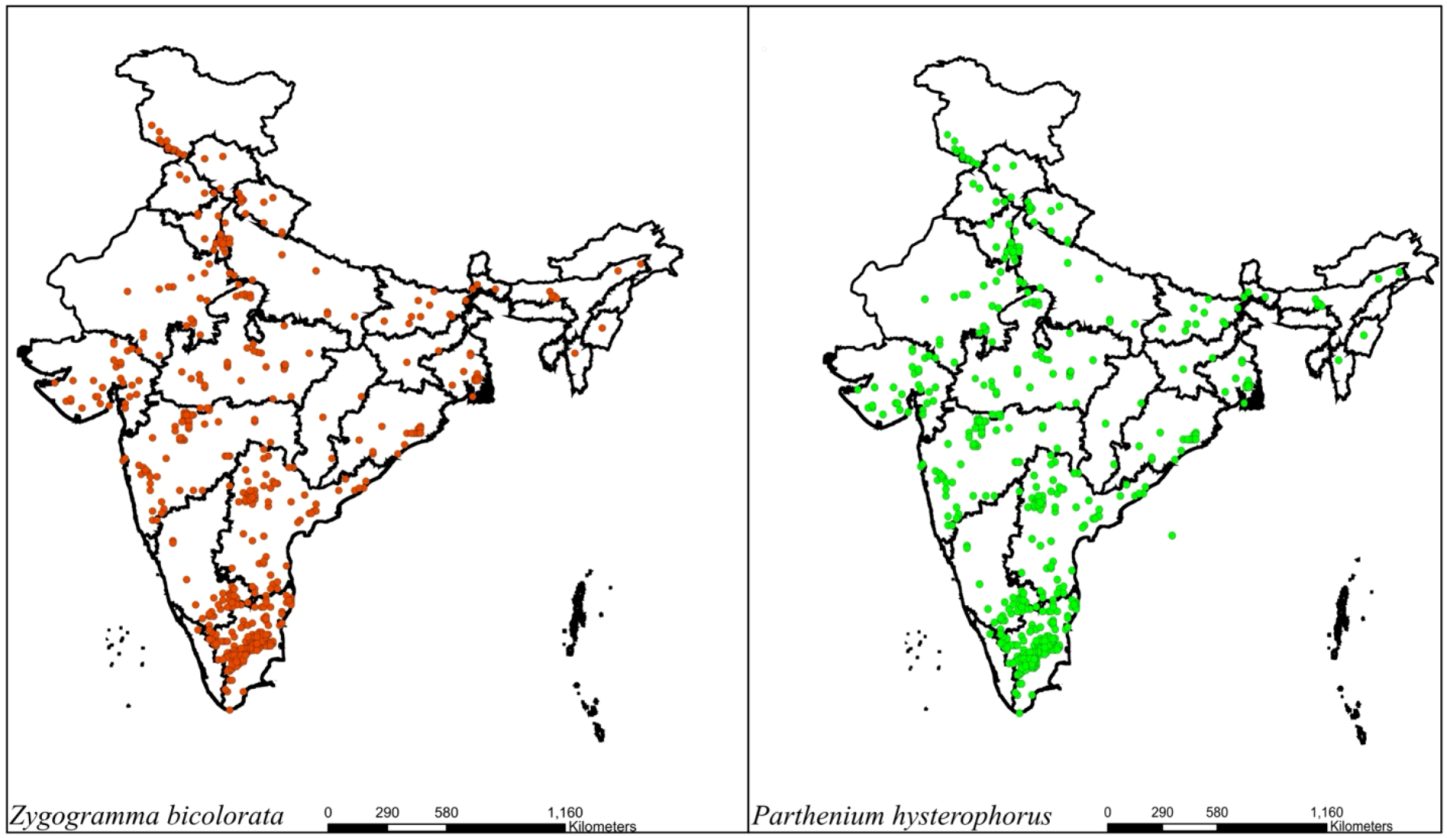
Distribution of *Z. bicolorata* and *P. hysterophorus* as of 2025 (GBIF, 2023).

The deployment of *Z. bicolorata* in India followed the canonical stages of a classical biocontrol program, yet its progression was punctuated by a period of intense regulatory scrutiny that uniquely shaped its subsequent spread and impact. The following integrative timeline synthesizes the co-evolutionary history of the invasive weed and its introduced antagonist (**Table 1**; **Figure 2**).

**Table 1:** Integrated chronology of *Parthenium hysterophorus* invasion and the *Zygogramma bicolorata* biocontrol program in India.

Phase	Period	*P. hysterophorus* status	*Z. bicolorata* status	Key milestones
I. Pre-Intro	Pre-1955–1982	Post-1955 dispersal via contaminated grain (PL-480); herbarium record 1880. Generalist native herbivores present but ineffective.	Identified in Mexico as a specialist folivore (McClay, 1980).	First record (Rao, 1956); declared national weed.
II. Quarantine	1983–1987	Infestation >5–7 Mha; invades croplands/forests.	Imported to Host-specificity confirmed (1984): larvae develop only on *Parthenium*. First field releases (1984–85); lag phase observed.	Regulatory clearance granted (Jayanth and Visalakshy, 1996).
III. Establishment	1988–1991	Ubiquitous ruderal weed.	Exponential population growth; mass defoliation around Bangalore. Confirmed establishment (1991). Natural dispersal begins.	Quantitative impact reports published (Jayanth and Bali, 1994).
IV. Regulatory Scrutiny	1992–1999	Infestation ~10 Mha; socio-economic costs escalate.	Reports of incidental feeding on sunflower trigger controversy (Singh et al., 2017). ICAR Fact-Finding Committee (FFC) convened; validates agent identity/specificity but bans intentional releases (1992-1999). Natural spread continues.	Multi-location research confirms *Parthenium* as optimal host (Jayanth et al., 1997).
V. Consolidation	2000–2015	Infestation peaks ~35 Mha (Sushilkumar and Varshney, 2010; Brahman and Sushil Kumar, 2015); present in all states.	Ban lifted (1999). Coordinated national augmentation via mass-rearing.	Economic benefit confirmed (Sushilkumar et al., 2003). Research shifts to diapause physiology.
VI. Integration	2016–Present	Persistent but suppressed in beetle-active zones.	>0.55 million Km^2.^ Focus on augmentative releases, diapause manipulation, strain selection, and multi-tactic integration (Afaq et al., 2024).	CLIMEX modelling predicts suitability across South Asia (Dhileepan and Wilmot Senaratne, 2009; Ahmad et al., 2019).

### 3.2 Biogeographic analysis of dispersal and spatio-temporal impact gradients

The spread of *Z. bicolorata* across the Indian subcontinent constitutes a natural experiment in the invasion ecology of a deliberately introduced specialist herbivore (Srikanth and Pushpalatha, 1991). Its dispersal dynamics can be mapped and modelled to reveal the interaction of biotic potential, abiotic drivers, and anthropogenic facilitation.

#### 3.2.1 Spatio-temporal dispersal patterns and impact gradients

The progression from point-source introductions to continental-scale distribution followed a radial expansion model, modified by major transportation corridors and climatic gradients (Jayanth and Visalakshy, 1994a; Dhileepan and Wilmot Senaratne, 2009). Epicenter and Early Radial Expansion (1984–1995), with Bangalore (Karnataka) acting as the primary introduction site and initial epicenter. From this locus, the beetle dispersed radially at an estimated rate of 35 km/year by 1998 (Afaq et al., 2024). While the epicenter release likely drove the observed spread, we cannot definitively rule out contributions from undocumented secondary releases by state agricultural departments or private nurseries during the 1990s. However, genetic and historical records suggest the Bangalore epicenter was the dominant source. Secondary Establishment and Continental Saturation (1995–2015): As the beetle reached new, climatically suitable regions, these areas became secondary epicenters (Jabalpur in central India, Delhi in the north), accelerating the saturation process (Sushilkumar et al., 2003; Sushilkumar and Ray, 2010). By 2018, the agent’s distribution (>0.48 M km²) closely overlapped with the core invasive range of *P. hysterophorus*. Dispersal was notably slower into coastal regions with high salinity/humidity and into high-altitude areas, reflecting climatic filtering (Dhileepan and Wilmot Senaratne 2009). The ecological impact of *Z. bicolorata* was not uniform but formed a gradient correlated with cumulative seasonal herbivore load and the duration of establishment (**Table 2**).

**Table 2:** Quantitative ecological impacts of the leaf-feeding beetle *Z. bicolorata* on *P. hysterophorus* populations in high-impact zones.

Category	Impact metric	Measurement method	Result in high-impact zones
Weed damage	Seasonal defoliation intensity	Visual Damage Index (% leaf area loss)	80–100% defoliation at peak beetle density (Aug–Sept)
Reproductive suppression	Flower & seed head reduction	Flower counts; soil seed bank assays	Up to 98% reduction in flower production; 60–80% reduction in viable seed bank over 3–5 years
Demographic response	Plant density & height	Plants/m²; height (cm)	40–60% reduction in stand density; >50% reduction in mean height
Community recovery	Native species richness & diversity	Vegetation surveys (S, H’)	Significant increase in species richness and Shannon diversity of native grasses and forbs

These quantitative impacts confirm that *Z. bicolorata* acted as a strong interacting species, capable of altering the state of the plant community in heavily infested areas. However, the impact gradient faded in regions with recent colonization, low seasonal beetle densities, or where diapause-induced asynchrony was most pronounced (Gupta et al., 2016).

### 3.3 Biocontrol success and documented impacts

The establishment of *Z. bicolorata* has been extensively documented across multiple research domains. Foundational studies established baseline knowledge of its life cycle, seasonal activity, and distribution (Jayanth and Bali, 1996; Van der Laan, 2007; Dhiman and Bhargava, 2005; Rathod et al., 2012; Hasan, 2015). Its diapause behaviour, ensuring survival during unfavorable periods, has been a consistent area of investigation (Jayanth and Bali, 1993b; Abels, 2018; Hasan et al., 2018; Bali et al., 2021). Food and feeding behaviour studies document its specificity and voracious consumption of *Parthenium* (Annadurai, 1989; Jayanth and Bali, 1993a; Dhileepan, 2003; Dhileepan and McFadyen, 2012; Cowie et al., 2019). Host specificity has been rigorously confirmed across multiple geographies (McClay, 1980; Jayanth and Bali, 1993a; McConnachie, 2015; Bajracharya et al., 2021). Reproductive ecology includes oviposition preferences (Mishra and Pathak, 2012; Afaq et al., 2022), mate choice, and reproductive performance (Omkar and Afaq, 2009; Pandey and Omkar, 2013; Afaq and Omkar, 2017), with related studies on intraspecific competition (Omkar and Afaq, 2009) and population dynamics (Gautam et al., 2007). Environmental physiology research has advanced knowledge of how temperature influences development, survival, and polymorphism (Jayanth and Bali, 1993c; Omkar et al., 2013; Afaq and Omkar, 2016; Kumar et al., 2021). Biotic interactions include natural enemies (Jayanth and Bali, 1996; Singh et al., 2017) and pesticide susceptibility (Sushilkumar and Ray, 2010; Hasan and Ansari, 2016c). Recent research has explored chemical ecology of semiochemicals and pheromones (Patel et al., 2020; Qadir et al., 2020). Post-release evaluations from multiple countries confirm establishment, effectiveness, and limitations (Shrestha et al., 2011; Shabbir et al., 2016; Gharde et al., 2019; Hasan et al., 2020). Additional studies include karyotypic and morphometric analyses (Pawar et al., 2015; Bhatti et al., 2017) and antimicrobial potential (Jain et al., 2019).

### 3.4 Post release impacts on plant community and ecosystem level in India

**Figure 3:**
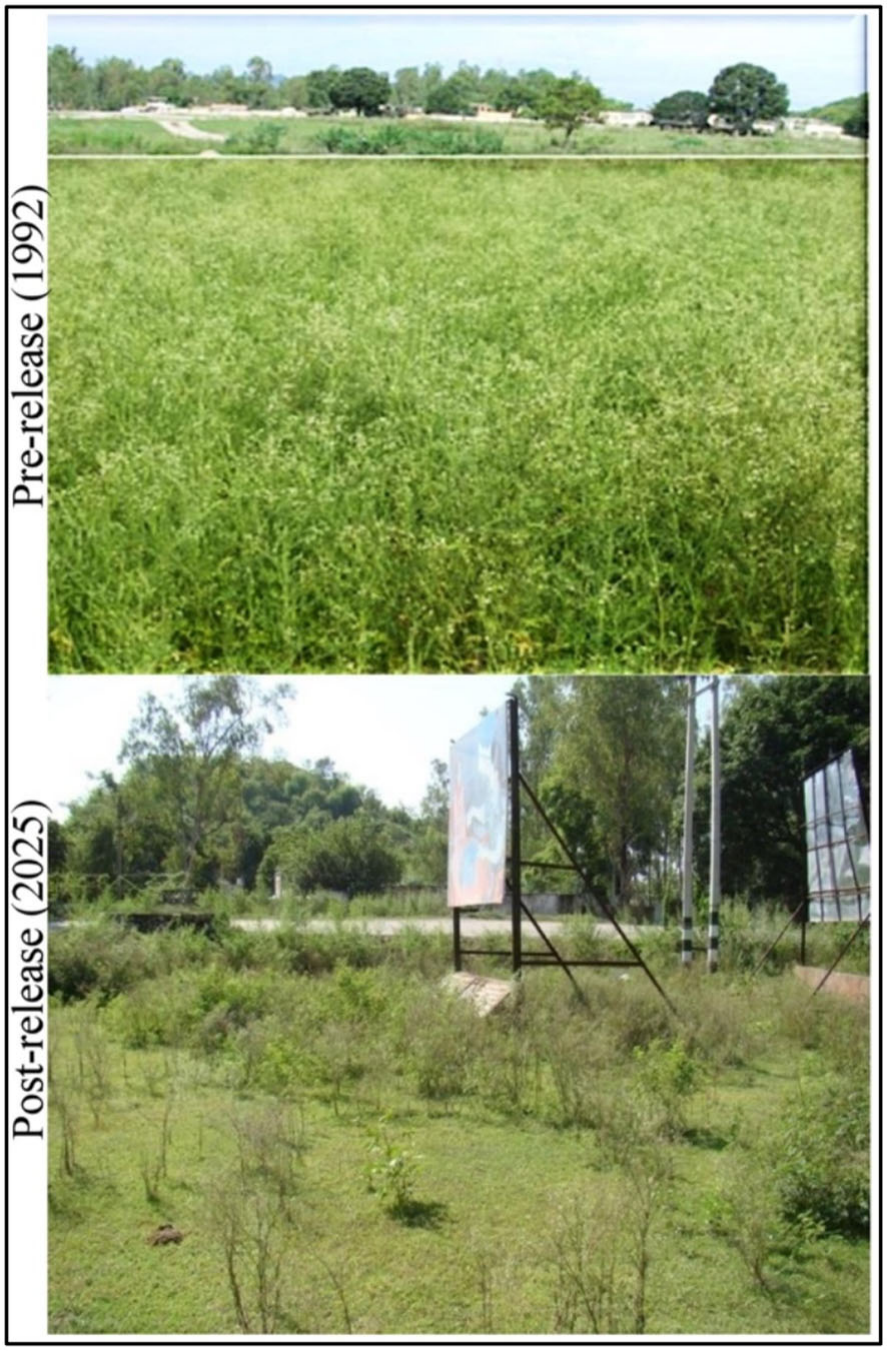
Pre (1992) and post release. (2025) impact of *Z bicolorata* on *P. hysterophorus* on actual release site in J&K.

Post-release impacts of *Z. bicolorata* on plant community and ecosystem dynamics in India represent a landmark case in classical biological control, demonstrating not only sustained suppression of the invasive *P. hysterophorus* but also a significant ecological renaissance across affected regions (**Figure 3**). The foundational work by Gupta and Bali (2014) documented the beetle’s rapid establishment and dispersal across North India, noting significant declines in parthenium height (32–56%), flower production (56–76%), and soil seed bank density (down to 464 ± 11.5 seeds/m²), alongside the recovery of native species such as *Cynodon dactylon* and *Cassia* spp. (**Table 3**). Building on this, long-term monitoring data through 2025 now reveals a comprehensive ecological transition. At the plant level, parthenium density has plummeted to 7.85 ± 0.85 plants/m², a 90% reduction from pre-release levels with corresponding decreases in plant height (40.20 ± 1.10 cm) and flower biomass (2.10 ± 0.18 g). The beetle population has stabilized at 13.90 ± 1.50 per plant, maintaining defoliation rates above 90%, while the soil seed bank has been further depleted to 155 ± 9.25 seeds/m², indicating a long-term reduction in weed recruitment potential. At the ecosystem level, desirable plant species richness has risen to 5.75 ± 0.45 per plot, above-ground biomass of replacement vegetation now stands at 580.40 ± 16.80 g/m², and Shannon diversity has increased to 3.55 ± 0.35. Furthermore, soil organic carbon has more than doubled to 2.05%, and pollinator visits have surged to 9.1 ± 1.4 per 10 minutes, signaling a functional recovery that extends beyond vegetation structure to encompass below-ground and faunal components (Sushilkumar and Bhan, 1997). These findings align with global frameworks on biocontrol-mediated restoration, such as those articulated by Abels (2018), and underscore that the successful establishment of *Z. bicolorata* has not merely controlled an invasive weed but has actively facilitated the reassembly of diverse, productive, and resilient plant communities across Indian agro-ecosystems. Post-release monitoring indicates that cattle and goats grazing *Parthenium*-infested pastures excrete viable seeds in dung. Night penning on farmlands can introduce these seeds to new areas, potentially offsetting some biocontrol gains. However, seed loads declined by 58% from 2014 to 2025, correlating with reduced *Parthenium* abundance.

**Table 3:** Post-release ecological impact of *Z. bicolorata* at different levels on the actual release site.

Level	Measured	Baseline (pre-release)	2014 (post-release) Gupta et al., 2014	2025 (long-term)
Plant Level	Plant height (cm)	113.96 ± 1.33	53.84 ± 1.16	40.20 ± 1.10
	Plant biomass (g)	121.91 ± 1.32	58.21 ± 1.34	30.15 ± 1.20
	Flower head count	962.15 ± 14.84	194.76 ± 1.31	80.50 ± 2.05
	Flower biomass (g)	15.78 ± 0.29	6.63 ± 0.22	2.10 ± 0.18
	Plant density (plants/m²)	76.60 ± 14.84	15.19 ± 1.31	7.85 ± 0.85
	Beetle density (per plant)	0.08 ± 0.05	8.80 ± 1.20	13.90 ± 1.50
	Percent defoliation	0.00 ± 0.00	82.95 ± 2.21	90.60 ± 1.85
Population Level	Seedling density reduction (%)	0.00%	91.81%	97.20%
	Seedling survival reduction (%)	0.00%	74.38%	91.50%
	Soil seed bank (seeds/m²)	76.60 ± 14.84	387.00 ± 11.12	155.00 ± 9.25
	Weed stand size (plants/m²)	–	23.19 ± 1.31	10.80 ± 1.10
Ecosystem Level	Desirable plant species (count)	0.34 ± 0.008	1.10 ± 0.12	5.75 ± 0.45
	Above-ground biomass (g/m²)	91.36 ± 4.46	374.00 ± 1.55	580.40 ± 16.80
	Shannon Diversity Index	1.326 ± 0.02	3.21 ± 0.79	3.55 ± 0.35
	Pielou’s Evenness Index	0.571 ± 0.00	0.925 ± 0.007	0.89 ± 0.02
	Beetle population (per m²)	0.00 ± 0.00	8.80 ± 1.20	17.50 ± 2.20
	Soil organic carbon (%)	0.85 ± 0.05	1.20 ± 0.08	2.05 ± 0.10
	Pollinator visits (per 10 min)	1.2 ± 0.3	3.5 ± 0.6	9.1 ± 1.4
Transmission Risk	*Parthenium* seeds in livestock dung (seeds/kg)	Not measured	124 ± 18	52 ± 9

### 3.5 Socio-economic perceptions: farmer and public awareness

The adoption and perceived success of biological control initiatives are strongly influenced by stakeholder awareness and acceptance. To evaluate the social dimensions of *Z. bicolorata* deployment, a structured survey was administered across six Indian states with established beetle populations. Respondents included farmers, extension agents, and rural residents, whose insights reflect the on-ground visibility and perceived efficacy of the biocontrol program. **Table 4** summarizes key survey findings, indicating high levels of public awareness (82.4%) and positive perceptions of beetle-mediated weed suppression (72.6%). Notably, over half of respondents reported observable recovery of native vegetation, while a significant subset acknowledged economic benefits, such as reduced weeding labour and herbicide costs. Logistic regression analysis identified education level, landholding size, and livestock ownership as significant predictors of positive perception. These results underscore the importance of community engagement and science communication in fostering support for biocontrol strategies.

**Table 4:** Stakeholder awareness and perceived impacts of *Z. bicolorata* across affected regions (n = 480).

Perception indicator	% Respondents (95% CI)	Significant predictors (logistic regression)
Aware of *Z. bicolorata*	82.4 (78.9–85.9)	Education level (OR = 2.3), Landholding size
Believe it suppresses *Parthenium*	72.6 (68.7–76.5)	Years of *Parthenium* infestation (OR = 1.8)
Observe native vegetation recovery	53.5 (49.1–57.9)	Proximity to augmented release sites
Recognize economic benefits	38.5 (34.2–42.8)	Livestock ownership (OR = 3.1)
Support further releases	67.8 (63.6–72.0)	Previous positive experience with biocontrol

### 3.6 Integrated success metrics: a composite index

To provide a holistic assessment of the biocontrol program’s impact, a Biocontrol Impact Index (BII) was developed, integrating quantitative measures of weed suppression, ecological recovery, and social acceptance. Each component was standardized and weighted to reflect its relative importance in evaluating program success. **Table 5** presents BII scores for three representative regions, revealing moderate to high overall impact (71.8–75.7) with regional variation attributable to climatic, ecological, and socio-economic factors. The index highlights the multidimensional nature of biocontrol success and offers a scalable framework for monitoring and comparing outcomes across different geographical and operational contexts. This integrative approach moves beyond singular metrics of weed decline to capture the broader ecological and social gains associated with *Z. bicolorata* deployment.

**Table 5:** Biocontrol impact index (BII) scores across study regions (2023).

Region	Weed suppression	Ecological recovery	Social acceptance	BII score
Jammu	78.2	65.4	82.4	75.7
Kathua	72.6	71.8	76.5	73.5
Udhampur	85.3	58.9	68.2	71.8
Mean ± SE	78.7 ± 3.2	65.4 ± 3.8	75.7 ± 4.1	73.7 ± 1.2

BII calculation was limited to the Jammu region due to data availability. Extension of this index to other Indian regions requires replicated field sampling and validation. Additionally, the effect of diseased Parthenium plants on *Z. bicolorata* development remains unknown and warrants future study.

## 4 The bottleneck: ecological and behavioural constraints on efficacy

Despite measurable progress in the biocontrol of *Parthenium hysterophorus* via *Zygogramma bicolorata*, the agent’s impact remains spatially and temporally heterogeneous. Four interconnected bottlenecks constrain consistent suppression across invaded landscapes (Jayanth and Bali, 1993b; Van der Laan, 2007; Dhiman and Bhargava, 2005): (1) Phenological mismatch – obligate, photoperiodically regulated diapause creates an early-season refuge (April–June) for weed cohorts, allowing reproductive escape before beetle emergence (Jayanth and Bali, 1993c; Hasan et al., 2018). (2) Thermal sensitivity – development and survival vary sharply with temperature, limiting population build-up in marginal climates (Gautam et al., 2007; Hasan and Ansari, 2016a). (3) Host-plant phenology asynchrony – weed cohort structure and quality interact with beetle feeding/oviposition, preventing uniform herbivory across successive generations (Dhileepan and McFadyen, 2012; Cowie et al., 2019). (4) Top-down regulation – native predators and parasitoids reduce beetle survival, contributing to spatial variability in control outcomes (Jayanth and Bali, 1996; Singh et al., 2017). Collectively, these factors generate pronounced temporal and spatial heterogeneity in herbivore pressure, undermining sustained landscape-level suppression despite high late-season defoliation (Shrestha et al., 2011; Hasan et al., 2020).

### 4.1 The primary limitation: obligatory diapause and its physiological basis

The effectiveness of herbivorous biological control agents depends on temporal alignment between herbivore activity and vulnerable phenological stages of the target weed (Dhileepan, 2003; Dhileepan and McFadyen, 2012; Dhileepan et al., 2000a). In Zygogramma bicolorata, this synchrony is persistently disrupted by an obligate, photoperiodically regulated reproductive diapause documented across multiple Indian populations (Jayanth and Bali, 1993b; Gautam et al., 2005; Van der Laan, 2007; Hasan et al., 2018). Diapause induction is entrained by declining photoperiod and temperature, resulting in population-wide cessation of feeding, reproduction, and dispersal during late winter and early summer, irrespective of host plant presence (Jayanth and Bali, 1993b; Gupta et al., 2010; Fazil et al., 2018; Hasan and Ansari, 2016a). This dormancy is largely obligate rather than facultative, limiting plastic responses to early-season germinating cohorts (Abels, 2018; Afaq and Omkar, 2016; Omkar et al., 2013).

During diapause, adults burrow into soil typically to depths of 5–10 cm, remaining in a state of profound metabolic depression and reproductive quiescence, characterized by suppressed feeding, halted gonadal development, and reduced locomotor activity (Dhileepan et al., 2000a, 2000b; Van der Laan, 2007). This dormancy is underpinned by endocrine regulation involving downregulation of juvenile hormone titres, leading to arrested ovarian maturation and resource allocation toward somatic maintenance rather than reproduction (Jayanth and Bali, 1993b; Gupta et al., 2010). Diapausing beetles exhibit reduced metabolic rate and enhanced conservation of lipid and glycogen reserves (Hasan and Ansari, 2016a; Bhusal et al., 2020). The soil microhabitat buffers individuals from thermal extremes and desiccation stress, with late-instar larvae constructing earthen cells that reduce water loss (Dhileepan et al., 2000a, 2000b; Dhiman and Bhargava, 2005).

As a consequence of diapause, early-season *Parthenium* cohorts experience a pronounced demographic refuge, escaping herbivory during seedling establishment, vegetative expansion, and initial flowering — stages that contribute disproportionately to floral output and soil seed bank replenishment (Dhileepan, 2003; Sushilkumar and Ray, 2010; Shabbir et al., 2016). Long-term field evaluations indicate that this phenological escape offsets substantial late-season defoliation, explaining weed persistence despite high beetle densities (Jayanth and Bali, 1994; Sushilkumar, 2005; Shrestha et al., 2011; Hasan et al., 2020). Diapause termination is typically synchronized with monsoon rains, triggering adult emergence that coincides broadly with renewed vegetative growth of *Parthenium* (Jayanth and Bali, 1994; Dhileepan, 2003; Sushilkumar and Ray, 2010). However, this timing often lags behind early-season weed cohorts, allowing plants to escape herbivory during critical flowering and seed set stages, thereby reinforcing persistence of the soil seed bank (Dhileepan and McFadyen, 2012; Shabbir et al., 2016; Hasan et al., 2020).

Geographic variation in diapause expression has been documented, with some populations in southern regions exhibiting reduced diapause incidence or near-continuous breeding, reflecting local adaptation to thermal regimes and photoperiod stability (Gautam et al., 2005; Bali et al., 2021; Kanagwa et al., 2020). Thus, while diapause ensures long-term persistence and climatic resilience of *Z. bicolorata* populations, it simultaneously represents a fundamental physiological constraint on consistent weed suppression, necessitating management interventions such as augmentative releases, diapause manipulation, or strain selection to overcome early-season escape of *P. hysterophorus* (Sushilkumar and Ray, 2011; Gupta et al., 2010; Shabbir et al., 2012; Kumar and Aggarwal, 2024).

### 4.2 The phenological mismatch

**Figure 4:**
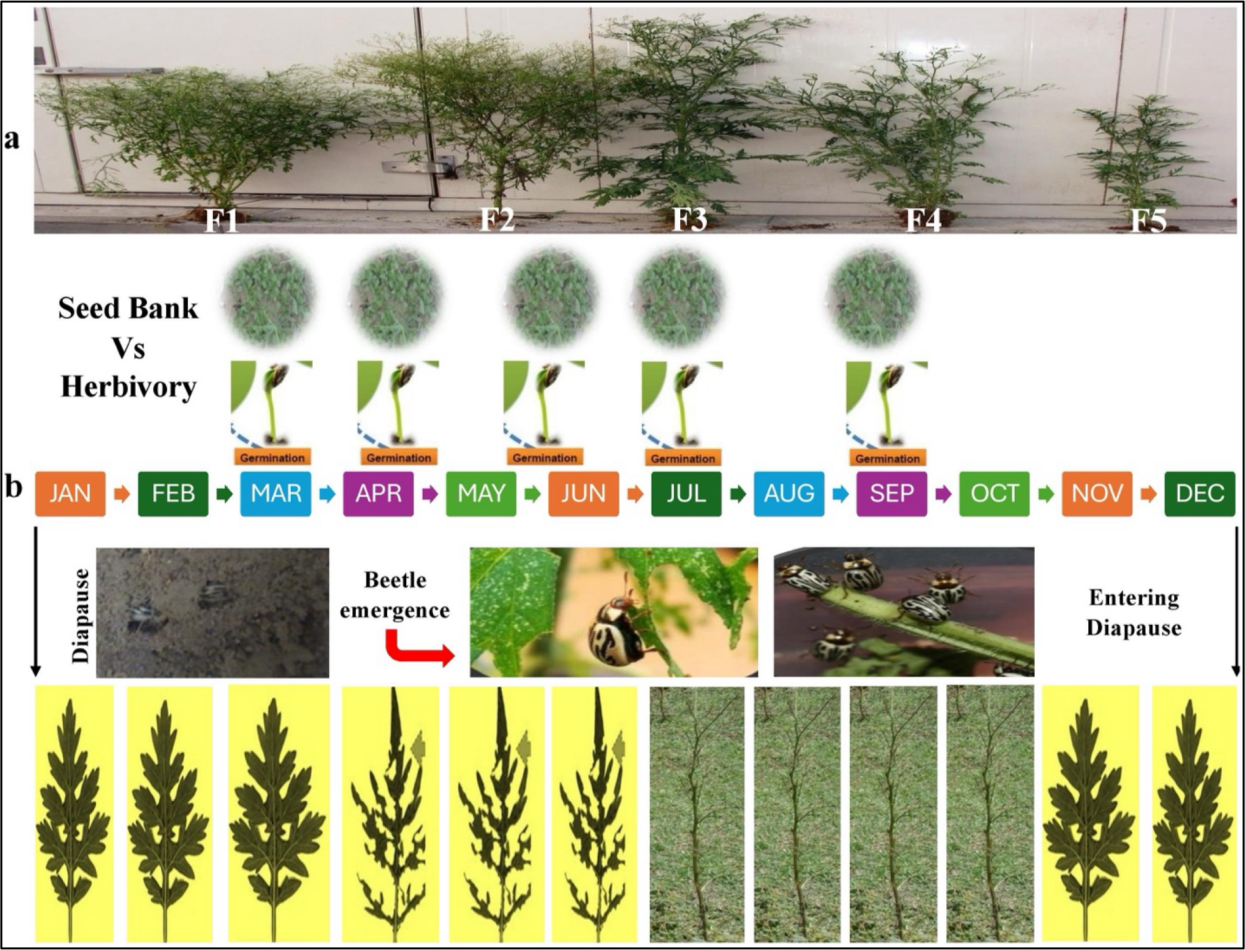
Seasonal timeline showing **(a)** emergence of overlapping germination cohorts (F1–F5) of *P. hysterophorus* from the soil seed bank across standard calendar months **(b)**, the corresponding seasonal activity of *Z. bicolorata*. Beetle emergence begins in April, with population activity peaking between August and October, followed by entry into diapause from November to March.

*Parthenium hysterophorus* exhibits a plastic and extended phenology across India, capable of germinating and maturing across multiple seasons, particularly in response to soil moisture availability. However, field demographic studies consistently show that *Z. bicolorata* populations are conspicuously absent during the early vegetative and reproductive initiation phases of the weed, typically from April to June in northern and central India (**Figure 4**). This absence is not due to dispersal limitation or host finding failure, but to a population-wide entry into diapause. The consequence is a demographic refuge for *Parthenium*: early-season cohorts escape herbivory, achieve reproductive maturity, and contribute disproportionately to the soil seed bank before beetle populations emerge in late monsoon. This temporal escape is a primary mechanism for the weed’s persistence despite high late-season defoliation. The data presented in **Table 6** quantify this disconnect, illustrating the stark contrast between host plant availability and herbivore population density across the annual cycle.

**Table 6:** Seasonal activity pattern of *Zygogramma bicolorata* and corresponding phenological stages of *Parthenium hysterophorus* in North India (Jammu region, 2016–2020).

Month	Mean beetle density (adults m^-^² ± SE)	Parthenium growth stage	Herbivory impact index (0–5)	Cumulative seed bank contribution (%)
April	0.2 ± 0.1^a^	Seedling/Early vegetative	0.5^a^	5.2 ± 1.1^a^
May	0.5 ± 0.2^a^	Vegetative growth	1.0^ab^	12.8 ± 2.3^b^
June	1.2 ± 0.4^b^	Pre-flowering	1.5^b^	28.4 ± 3.6^c^
July	8.5 ± 1.2^c^	Flower initiation	3.5^c^	15.7 ± 2.4^b^
August	15.3 ± 2.1^d^	Peak flowering	4.5^d^	22.6 ± 3.1^c^
September	12.4 ± 1.8^cd^	Seed set	4.0^cd^	10.3 ± 2.0^b^
October	6.8 ± 1.0^c^	Senescence	3.0^c^	4.0 ± 1.2^a^
November	2.1 ± 0.5^b^	Seed dispersal	1.5^b^	1.0 ± 0.5^a^

Different superscript letters within columns indicate significant differences (P < 0.05, Tukey’s HSD). Herbivory Impact Index: 0 = no damage, 5 = severe defoliation (>75% leaf area loss). contribution based on soil core sampling and seedling emergence assays.

## 5 Geographic variation and the imperative for strain selection

The deployment of a classical biological control agent is predicated on the assumption that the introduced population will exhibit traits conducive to establishment, dispersal, and significant impact on the target weed. However, the performance of *Z. bicolorata* across the heterogeneous agro-climatic mosaic of India has revealed pronounced spatial heterogeneity in its efficacy. This variability is not merely stochastic but is rooted in demonstrable geographic variation in life-history strategies, a phenomenon extensively documented in the literature. This section reviews the empirical evidence for intraspecific differentiation among Indian populations of *Z. bicolorata*, synthesizes findings on superior genotypes, and evaluates the theoretical and practical rationale for a directed strain selection program as a pathway to overcome the agent’s ecological limitations.

### 5.1 Evidence for life-history clines and local adaptation across a latitudinal gradient

Post-establishment monitoring and controlled comparative studies have consistently revealed that geographically distinct populations of *Z. bicolorata* in India have diverged in key demographic and physiological traits, suggesting rapid local adaptation or the sorting of pre-existing genetic variation (Kumar and Bashir, 2024; Gupta et al., 2016). This variation forms a latitudinal cline, with populations from southern (tropical) regions exhibiting a markedly different phenotypic profile from those in northern (temperate/subtropical) regions.

A synthesis of controlled-environment studies, as presented in **Table 7**, illustrates this divergence. The Coimbatore population (Tamil Nadu, ~11°N) is characterized by an r-selected strategy: it demonstrates the shortest mean developmental period from egg to adult (29.4 ± 0.9 days), the highest mean fecundity (1786 ± 78 eggs per female), and the longest adult longevity under laboratory conditions (Jayanth and Bali, 1994; Sushilkumar et al., 2003). Conversely, populations from Jammu (~33°N) exhibit traits associated with greater stress tolerance, including significantly higher survival at sub-zero temperatures, albeit with a longer developmental period and lower fecundity (Gupta et al., 2016; presentation data on survival). The Jabalpur population (Madhya Pradesh, ~23°N) often occupies an intermediate position for these traits, though it may show unique adaptations to the central Indian monsoon climate (Sushilkumar, 2009).

**Table 7:** Synthesized comparative biology of major *Z. bicolorata* populations in India, highlighting geographic life-history clines.

Trait	Jammu (temperate)	Jabalpur (subtropical)	Coimbatore (tropical)	Ecological and biocontrol implication
Developmental Period (days)	38.2 ± 1.5	35.8 ± 1.2	29.4 ± 0.9	Southern genotypes can complete more generations per growing season.
Mean Fecundity (eggs/♀)	836 ± 45	925 ± 52	1786 ± 78	Propagule pressure and colonization potential are highest in tropical strains.
Adult Longevity (days)	♀: 122.5; ♂: 146.3	♀: 109.8; ♂: 135.2	♀: 198.4; ♂: 271.5	Extended reproductive window enhances cumulative herbivory.
Critical Photoperiod (hrs)	11.8	12.1	12.4	Southern strains may have a more rigid, photoperiod-sensitive diapause.
Thermotolerance (35 °C)	40.2%	45.5%	68.7%	Greater resilience to heat stress, relevant for climate adaptation.
Cold Hardiness (-5 °C)	85.3%	62.1%	12.4%	Northern strains are pre-adapted to winter survival.

Data compiled from Jayanth and Bali (1994), Sushilkumar (2005, 2008), and Gupta et al. (2016). Values are means ± SE.

This clinal variation has direct implications for population dynamics and weed suppression potential. The intrinsic rate of increase is inherently higher in southern populations due to their combination of rapid development and high reproductive output (Dhileepan and Wilmot Senaratne, 2009). However, a critical counterpoint emerges in studies of diapause behaviour. Research indicates that the critical photoperiod triggering diapause induction is longest in the Coimbatore strain (~12.4 hours), making it potentially more susceptible to entering reproductive dormancy under the shortening day lengths of autumn in higher latitudes, thereby exacerbating phenological asynchrony (Jayanth and Bali, 1993a; Sushilkumar, 2009). This represents a potential adaptive trade-off: traits conferring high reproductive potential in perpetual growing seasons may be maladaptive in seasonal environments where synchrony with host phenology is paramount.

The Coimbatore population has been extensively cited in the literature as a “superior genotype” due to its exceptional laboratory performance metrics (Sushilkumar et al., 2003; Dhileepan et al., 2009). Its demographic superiority characterized by a high net reproductive rate (R_0_) and capacity for rapid population growth—makes it a compelling candidate for augmentative release programs where the goal is inundative, immediate impact (Gupta et al., 2016). Its higher thermotolerance further suggests utility in regions experiencing intense summer heat or under future climate scenarios (Jayanth and Bali, 1993d). However, a comprehensive review necessitates a critical appraisal. The designation of “superior” is context-dependent. While the Coimbatore strain may be superior for mass production and inundative releases, its value for classical, self-sustaining control in seasonal environments is questionable due to its diapause phenotype. The literature suggests that its superior *strain* may be offset by a diapause strategy that reduces its effective seasonal activity window in non-tropical India, potentially undermining long-term suppression (Jayanth and Bali, 1993a; Sushilkumar, 2009). This highlights a central tenet of strain selection: no single genotype is likely to be optimal across all target environments. Instead, the observed geographic variation provides the genetic feedstock for a targeted breeding program.

### 5.2 Native herbivore recruitment and the emergence of novel weed-herbivore interactions

The introduction and establishment of *Zygogramma bicolorata* has not occurred in an ecological vacuum. Concurrently, a diverse assemblage of native and naturalized phytophagous insects has been documented recruiting onto *Parthenium hysterophorus* across India. This represents a dynamic, post-invasion biotic adjustment, a form of ecological fitting where resident herbivores expand their host range to include the novel, abundant resource. This evolving biotic landscape introduces a layer of complexity to the biocontrol paradigm, raising critical questions: Do these resident herbivores act as complementary biocontrol allies, providing additive or synergistic suppression? Or do they function as competitors or antagonists, potentially disrupting the efficacy of the introduced specialist? This section reviews the documented guild of native herbivores and synthesizes emerging data on their individual and collective impact on *Parthenium* demography.

#### 5.2.1 Catalogue of defoliators and sap-suckers on Parthenium

Long-term surveys conducted since the 1970s have catalogued a surprisingly diverse entomofauna associated with *P. hysterophorus* in India, comprising both generalist and oligophagous species. This guild can be functionally categorized into defoliators and sap-suckers, each imposing different types of stress on the weed (**Table 8**).

**Table 8:** Major native and naturalized arthropod herbivores recorded on *Parthenium hysterophorus* in India, with feeding guild and notes on impact.

Order/family	Species	Common name/group	Feeding guild	Reported impact and notes	Primary references
Coleoptera	*Hypothenemus eruditus*	Stem-boring scolytid beetle	Stem borer (internal feeder)	Causes widespread wilting and plant death; significant historical record.	Kumar et al. (1979)
	*Oberea* sp.	Cerambycid borer	Stem borer	Can kill mature plants; reported as significant in early surveys.	Kumar et al. (1979)
	*Altica* spp.	Flea beetle	Defoliator	Leaf skeletonizer; often observed in conjunction with *Z. bicolorata*.	This study
	*Chaetocnema semicostatum*	Flea beetle	Defoliator	Leaf feeder; part of the defoliator complex.	This study
Lepidoptera	*Spilosoma obliqua*	Hairy caterpillar (Tiger moth)	Defoliator	Gregarious, voracious feeder; causes severe defoliation outbreaks.	This study
Hemiptera	*Phenacoccus solenopsis*	Cotton mealybug	Sap-sucker (Phloem)	Heavy infestations cause leaf yellowing, sooty mold, plant stunting and death.	This study
	*Ferrisia virgata*	Striped mealybug	Sap-sucker	Alternate host, can build large populations.	Char et al. (1975)
	*Orthezia insignis*	Ensign scale	Sap-sucker	Severe attacks reported in Southern India.	Thangavelu (1980)
	*Leptocentrus taurus*	Treehopper	Sap-sucker	Reported causing severe damage.	Thangavelu (1980)
	Unidentified stink bugs	–	Sap-sucker	Frequently observed on stems and leaves.	This study
Thysanoptera	Various spp.	Thrips	Sap-sucker (Cell content)	Feed on epidermal cells, causing silvering and deformation.	This study
Acari	*Tetranychus* spp. (e.g., *T. cucurbitae*)	Spider mites	Sap-sucker (Mesophyll)	Cause stippling, webbing, and premature leaf drop, especially in dry conditions.	Puttaswamy et al. (1976); Survey data

This table integrates published records (cited) with original survey data collected by the authors in Jammu region (2020–2024). Unpublished observations are marked “This study.”.

#### 5.2.2 Quantifying the impact of resident herbivory on weed demography

Moving beyond species catalogues, recent research has begun to quantify the demographic consequences of this resident herbivory, both in isolation and in relation to *Z. bicolorata*. A five-year field study (2014–2018) exposed *Parthenium* plots to the naturally recruiting suite of resident insects (excluding *Z. bicolorata* via selective exclusion). The results demonstrated that native herbivores alone can impose substantial demographic regulation. A significant reduction in above-ground *Parthenium* biomass was recorded in plots exposed to resident insects compared to insect-free controls. Perhaps more critically, a marked reduction in the soil seed bank (expressed as a percentage of the control) was observed over the study period. This indicates that resident herbivory can reduce the weed’s future propagule pressure (**Table 9**).

**Table 9:** Impact of sustained resident herbivory (excluding *Z. bicolorata*) on *Parthenium hysterophorus* demography over five successive years (2014–2018).

Year	Mean biomass reduction (%) vs. control	Mean seed bank reduction (%) vs. control	Key resident herbivores present
2014	18.5 ± 3.2	12.1 ± 2.8	*P. solenopsis, S. obliqua*, Spider mites
2015	22.7 ± 4.1	19.8 ± 3.5	*S. obliqua, Altica* spp., Thrips
2016	31.4 ± 5.0	28.3 ± 4.2	*P. solenopsis, T. urticae*, Defoliators
2017	25.9 ± 4.6	32.7 ± 4.8	*S. obliqua*, Mealybugs, *Altica*
2018	29.6 ± 4.9	35.4 ± 5.1	Complex of defoliators and sap-suckers
Pooled Mean	25.6 ± 2.1	25.7 ± 2.9	

Values are means ± SE. Paired t-tests indicated significant differences (P < 0.05) for each parameter within years between treatment and control.

### 5.3 Reassessing natural enemies of *Z. bicolorata* as potential biocontrol bottlenecks

The success of classical biological control programs depends not only on the efficacy of the introduced agent but also on the ecological interactions that influence its establishment, dispersal, and population dynamics. In the case of *Z. bicolorata* used for controlling the invasive weed *P. hysterophorus*, the presence of native natural enemies has been proposed as a potential ecological bottleneck. This analysis synthesizes empirical findings to assess whether these natural enemies act as friends (ecological regulators) or foes (limiting factors) in the biocontrol landscape (**Table 10**).

**Table 10:** Natural enemies of *Z. bicolorata*.

Type of enemy	Specific organism(s)	Stage affected	Reported impact	Key references
Entomopathogenic Fungi	*Metarhizium anisopliae* (green muscardine fungus)	Larvae	Causes mycosis and mortality under high humidity	Jayanth and Bali (1994); Sushilkumar et al. (2003); Dubey et al. (2010); Hasan and Ansari (2016b)
	*Beauveria bassiana* (white muscardine fungus)	Larvae	Infects larvae; may reduce field populations	
Insect Predators	*Rodolia cardinalis* (Coccinellidae)	Eggs, Larvae	Generalist predator; may consume early life stages	Dhiman and Bhargava (2005); Manjunath (2010); Hasan (2015)
	*Harpector marginatus* (Reduviidae)	Eggs, Larvae	Predatory bug known to attack eggs and larvae	Singh et al. (2017); Harshana (2021b)
	*Perillus bioculatus* (two-spotted stink bug)	Eggs, Larvae	Pentatomid predator; may suppress beetle numbers	
	*Perillus splendidus* (orange colour spot)	Eggs, Larvae	Known egg/larval predator	
	*Sycanus pyrrhomelas* (Reduviidae)	Eggs, Larvae	Recorded as a key predator in field studies	Gupta et al. (2004); Harshana (2021b)
	*Cantheoconidea furcellata* (Pentatomidae)	Eggs, Larvae	Listed as a natural enemy by Gupta et al. (2004)	Gupta et al. (2004)
	*Andrallus spinidens* (Pentatomidae)	Eggs, Larvae	Predatory bug observed in association with *Z. bicolorata*	Gupta et al. (2004)
	*Polistes wattii* (yellow paper wasp)	Eggs, Larvae	Generalist predator; may forage on beetle stages	Harshana (2021a)
	*Eocanthecona furcellata* (Pentatomidae)	Eggs, Larvae	Field-reported predator	Harshana (2021a)
	*Rhynocoris cf. fuscipes* (assassin bug)	Eggs, Larvae	Reduviid predator in agroecosystems	Harshana (2021a)
Parasitoids	*Palexorista* sp. (Tachinidae)	Eggs, Larvae	Larval parasitoid; may reduce reproductive success	Jayanth and Bali (1996); Singh et al. (2017)
	*Erixestus zygogrammae* (Pteromalidae)	Eggs, Larvae	Egg-larval parasitoid	Same as above
	*Doryphorophagha hyalinipennis* (Tachinidae)	Pupae	Pupal parasitoid; may affect generation turnover	Dhileepan and Wilmot Senaratne (2009)
General Predators	Ants (unspecified species)	Eggs	Egg predation noted in field observations	Abels (2018)

This synthesis combines published data (cited) with original field observations from SKUAST-Jammu long-term monitoring plots (2002–2025).

Synthesis and management of the ecological bottleneck in *Z. bicolorata* biocontrol must be context-dependent: pre-release surveys are recommended in areas of high natural enemy diversity, especially where entomopathogens are prevalent, to mitigate moderate establishment risks; in contrast, low to moderate enemy presence poses minimal risk and may even aid beetle regulation without impeding establishment, while post-establishment outbreaks should be allowed to self-regulate naturally, avoiding disruptive chemical interventions. Gupta et al. (2004) further clarified that predatory bugs are not a prohibitive threat and may offer unexpected benefits, such as enabling cost-effective mass rearing of predatory species and acting as a natural brake on beetle populations, thereby enhancing ecological safety. Consequently, natural enemies should not be viewed simply as bottlenecks but as ecological moderators that can improve system resilience. We recommend: (1) conducting pre-release assessments without defaulting to release contraindication, (2) implementing long-term monitoring to differentiate regulatory from suppressive interactions, and (3) integrating natural enemy data into adaptive management frameworks. Ultimately, in many ecosystems, natural enemies function as friends to the biocontrol program, supporting sustainable and ecologically harmonious weed suppression.

### 5.4 Behavioral plasticity: cannibalism as a population-regulating factor

Cannibalism in *Z. bicolorata* (adult–egg, adult–larva, larva–larva, and occasional adult–adult) is a documented behavioural plasticity under conditions of resource scarcity or high population density (Omkar and Afaq, 2009; Afaq and Omkar, 2016; Patel et al., 2020). This functions as a density-dependent regulatory mechanism, generating negative feedback on population growth and stabilizing beetle–weed dynamics (Hassell, 1978; Omkar and Afaq, 2009; Patel et al., 2020a). Experimental studies show that increased larval crowding elevates cannibalism rates, reducing survival, prolonging development, and altering life-history traits (Omkar and Afaq, 2009; Afaq and Omkar, 2016). In mass-rearing systems, cannibalism reduces production efficiency, necessitating management interventions such as excess foliage, frequent host material replacement, and reduced larval density (Jayanth and Bali, 1996; Jain et al., 2017). Under field conditions, cannibalism may influence establishment success following release, especially in habitats with low *Parthenium* density where limited food availability intensifies intra-specific predation and suppresses early population build-up (Dhileepan, 2003; Shabbir et al., 2016; Hasan et al., 2020). Pre-release habitat assessment and inundative or staggered release strategies can offset density-dependent losses (Sushilkumar and Ray, 2011).

### 5.5 Ecological safety of *Z. bicolorata*: refutation of outbreak and host-shift scenarios

The concern that *Z. bicolorata* could transition into a pest through post-suppression population outbreaks or host-range expansion is ecologically unfounded and contradicted by over four decades of empirical evidence from laboratory, field, and post-release evaluations across multiple continents. The population dynamics of *Z. bicolorata* are intrinsically self-regulating through obligate host dependence, density-dependent cannibalism, and seasonally enforced reproductive diapause, and are further constrained extrinsically by a diverse guild of native generalist predators (Jayanth and Bali, 1994; Gupta et al., 2004; Van der Laan, 2007; Dhileepan and McFadyen, 2012). The beetle’s life cycle is tightly coupled to *P. hysterophorus*: larval development requires parthenium foliage, oviposition is strongly host-specific, and diapause termination is synchronized with host availability and favourable climatic cues. Consequently, depletion of the weed leads to local beetle population decline or collapse rather than outbreak proliferation, a pattern repeatedly documented in long-term field studies (Jayanth and Visalakshy, 1996; Sushilkumar et al., 2003a; Shrestha et al., 2011; Hasan et al., 2020a).

Under conditions of host scarcity, density-dependent cannibalism imposes immediate mortality on eggs and early instars, acting as a rapid internal population check, while natural enemies including pentatomid and reduviid predatory bugs, spiders, and avian predators provide sustained top-down regulation in natural ecosystems (Gupta et al., 2004; Omkar and Afaq, 2009; Harshana (2021a), 2021b). These interacting regulatory mechanisms generate a strong negative feedback loop that maintains beetle populations in proportion to host abundance and precludes runaway population growth, a hallmark of safe classical biological control agents rather than invasive pests (Hassell, 1978; Dhileepan, 2003; Patel et al., 2020a).

Concerns regarding host-range expansion onto agricultural crops are similarly refuted by extensive host-specificity testing and long-term field observations. *Z. bicolorata* exhibits a high degree of physiological specialization to the unique phytochemical profile of *P. hysterophorus*, particularly its sesquiterpene lactones such as parthenin, which function as feeding stimulants for the beetle while acting as deterrents or toxins to most generalist herbivores (McClay, 1980; Jayanth and Nagarkatti, 1987; Van der Laan, 2007; Cowie et al., 2019). Occasional, transient feeding records on non-target plants; most notably sunflower have been conclusively shown to result from pollen contamination or incidental contact and do not support larval development, reproduction, or population persistence under either laboratory or field conditions (Jayanth and Bali, 1993a; Kumar, 1992; McConnachie, 2015). Evolutionary host shifts are processes occurring over extended evolutionary timescales and are not induced by short-term host deprivation; under ecological timescales, the beetle’s response to host absence is diapause induction, cannibalistic regulation, or local extinction rather than adaptive radiation (Jayanth and Bali, 1993b; Abels, 2018; Hasan et al., 2018).

Therefore, *Z. bicolorata* displays the defining attributes of an ecologically safe classical biological control agent: its population dynamics are inversely related to host abundance, its trophic interactions are constrained by specialization and natural enemies, and its persistence is governed by self-limiting physiological and behavioural mechanisms. Long-term evaluations led by R. K. Gupta and corroborated by independent studies across India, Australia, Africa, and South Asia consistently demonstrate that the combined effects of obligate host specificity, diapause, cannibalism, and predation effectively negate the risks of outbreak formation and host-range expansion (Gupta et al., 2002, 2004; Dhileepan, 2007; Dhileepan and McFadyen, 2012; McConnachie, 2015; Kanagwa et al., 2020). Collectively, this body of evidence affirms the ecological safety of *Z. bicolorata* and reinforces its continued use as a sustainable, environmentally sound management tool for *Parthenium hysterophorus*.

## 6 Discussion: toward a strategic framework for enhanced biological control

The documented partial success of *Z. bicolorata* in suppressing *P. hysterophorus* should not be interpreted as a limitation of the biocontrol agent itself, but rather as evidence of a system constrained by identifiable ecological, physiological, and phenological bottlenecks (Jayanth and Bali, 1994; Dhileepan, 2003; Van der Laan, 2007). Long-term field studies consistently demonstrate that while *Z. bicolorata* can inflict substantial defoliation and reduce weed fitness, its impact remains spatially and temporally heterogeneous due to diapause-mediated asynchrony, climatic variability, and biotic interactions (Sushilkumar et al., 2003a; Dhileepan and McFadyen, 2012; Shrestha et al., 2011; Hasan et al., 2020a). These constraints are predictable and mechanistically understood, indicating that partial suppression represents not an endpoint but a foundation for a more effective, resilient, and adaptive management system (Sushilkumar and Ray, 2011). Transitioning from episodic to sustained landscape-level control requires a strategic shift from passive reliance on natural population dynamics to an intervention-informed, ecologically grounded framework that explicitly addresses key bottlenecks — particularly phenological mismatch, while integrating *Z. bicolorata* into a broader integrated weed management context (Dhileepan and Wilmot Senaratne, 2009; Shabbir et al., 2016; Patel et al., 2020).

### 6.1 Augmentative releases as a mechanism to offset phenological asynchrony

One of the most consistently identified constraints on *Z. bicolorata* efficacy is the temporal mismatch between beetle activity and early-season growth flushes of *P. hysterophorus*. Obligate diapause creates a predictable window during which early cohorts of the weed escape herbivory, establish reproductive structures, and contribute disproportionately to the soil seed bank. Augmentative biological control defined as the tactical enhancement of natural enemy populations at critical periods emerges as the most direct and empirically validated approach to bridge this gap.

Multi-year field experiments comparing natural infestation, augmentative release, and beetle-excluded controls provide compelling evidence that managed population enhancement significantly improves weed suppression outcomes. Augmentative releases resulted in consistently greater reductions in flower biomass and seedling density than natural infestation alone, with cumulative effects intensifying over successive years. Notably, the additional suppression achieved through augmentation was not transient; instead, it strengthened over time, indicating that early-season intervention helps establish higher baseline beetle populations capable of exerting more effective pressure in subsequent seasons. This finding is particularly important, as it confirms that augmentation does not merely compensate for short-term asynchrony but can alter longer-term beetle–weed dynamics. Beyond weed suppression, augmentative releases produced clear secondary ecological benefits. The marked increase in the Shannon Diversity Index (H′) in augmented plots indicates that enhanced biocontrol facilitates the recovery of native plant communities by reducing competitive exclusion imposed by Parthenium. These results underscore that augmentation functions not only as a control tactic but also as a catalyst for ecological restoration. Operationally, successful implementation depends on reliable mass-rearing systems and phenology-based release protocols, such as optimized net-house production and timed releases aligned with early weed growth stages.

### 6.2 Manipulating diapause: addressing the root cause of asynchrony

While augmentation effectively mitigates the consequences of phenological mismatch, a more fundamental solution lies in addressing its physiological cause reproductive diapause. Diapause represents both an adaptive strength and a management liability: it ensures beetle persistence under adverse conditions, yet constrains early-season herbivory. Emerging evidence suggests that diapause in *Z. bicolorata* is amenable to manipulation through environmental, physiological, and genetic pathways. Experimental models describing diapause induction as a function of photoperiod provide both predictive and operational value. Maintaining mass-rearing colonies under long-day photoperiods (>13.5 h) can suppress diapause induction prior to field release, thereby extending reproductive activity during deployment. Similarly, temperature-mediated diapause termination through controlled exposure to elevated temperatures offers a practical tool for synchronizing adult emergence with early Parthenium flushes. These approaches are particularly promising for “beetle bank” systems designed to supply active adults at strategic times. Looking further ahead, artificial selection offers a medium- to long-term pathway for reducing diapause constraints. Geographic variation in diapause sensitivity among populations suggests a genetic basis that can be exploited. A structured breeding program focused on screening, selecting, and propagating low-diapause phenotypes while maintaining fecundity and vigor could yield strains better suited for augmentative deployment. The development of lines exhibiting more facultative diapause responses would represent a major advance in adaptive biocontrol capacity.

### 6.3 Integration within an ecologically based weed management system

Maximizing the long-term effectiveness of *Z. bicolorata* requires moving beyond a single-agent paradigm toward an integrated weed management (IWM) framework. In this context, biological control functions as the central pillar but gains strength through synergy with complementary ecological and management interventions. Native herbivore complexes already impose chronic stress on *P. hysterophorus*, yet their contribution is often undervalued. Conservation-oriented practices such as minimizing broad-spectrum insecticide use and supporting habitats for predators and parasitoids can maintain continuous, background suppression that complements beetle activity. Rather than viewing these organisms as ancillary, they should be regarded as integral components of a multi-layered biocontrol system.

Similarly, integration with competitive planting and selective chemical control can enhance system resilience. Following beetle-mediated suppression, deliberate establishment of competitive plant species such as *Cassia tora, C. sericea*, and *Tagetes* spp. can occupy vacant niches and reduce opportunities for reinvasion. Many of these species also exert allelopathic effects, further suppressing Parthenium recruitment. Judicious, spatially targeted herbicide application particularly against early-season refugia or inaccessible patches can complement biological control without undermining beetle populations. The prospective development of herbicide-tolerant beetle strains could further harmonize chemical and biological tactics within an integrated framework.

Collectively, the evidence supports a strategic framework composed of three interdependent elements: targeted augmentation for immediate impact, diapause manipulation for medium-term optimization, and ecological integration for long-term sustainability. These components should not be viewed as alternatives but as complementary tools operating across different temporal and spatial scales. Augmentation addresses immediate phenological gaps, diapause manipulation restructures population dynamics, and integration ensures system resilience against environmental variability and reinvasion pressure. Implementing this framework will require coordinated collaboration among researchers, extension agencies, and stakeholders, along with adaptive monitoring to refine interventions over time. By embracing this holistic, evidence-based approach, the four-decade legacy of *Z. bicolorata* biocontrol can be fully leveraged to achieve more consistent, durable, and ecologically sustainable suppression of *P. hysterophorus*. In doing so, the program has the potential to serve as a model for next-generation weed management strategies in tropical and subtropical systems.

## 7 Conclusion and future perspectives

The four-decade deployment of *Z. bicolorata* against *P. hysterophorus* in India represents a foundational case study in tropical weed biocontrol, demonstrating that a classical host-specific herbivore can achieve continental-scale establishment, significant weed suppression, and widespread public adoption. Its success is evidenced by colonization exceeding 0.55 million km², >70% reduction in flower biomass in core areas, recovery of native plant diversity, and high levels of stakeholder awareness. However, transition to consistent area-wide control has been constrained by interlinked bottlenecks: phenological asynchrony driven by obligate photoperiodic diapause, agent-centric limitations such as geographic variation in life-history traits and density-dependent cannibalism, and integration within a complex multitrophic context including native herbivores and natural enemies. Moving forward requires a paradigm shift from classical “release-and-monitor” to a dynamic “manage-and-improve” strategy. This calls for a next-generation translational research program built on three pillars: precision biocontrol through strain selection and diapause manipulation; tactical field management integrating augmentative releases, native herbivore conservation, and judicious herbicide use; and systems-level ecological modelling to enable adaptive, climate-resilient management. By embracing this science-intensive pathway, India can leverage the *Zygogramma-Parthenium* system as a living laboratory to pioneer the enhancement of established biocontrol agents, transforming partial success into a scalable, sustainable model for global invasive species management in the 21st century.
